# Do we react differently toward bionic devices vs. cochlear implants and wheelchairs? Possible links with personality traits

**DOI:** 10.3389/fresc.2023.1159663

**Published:** 2023-07-11

**Authors:** Diana-Alina Oancea-Matei, Alois Gherguț, Alexandra Maftei

**Affiliations:** Department of Education Sciences, Faculty of Psychology and Education Sciences, Alexandru Ioan Cuza University, Iași, Romania

**Keywords:** disability, personality, bionic eye, bionic limb, attitudes

## Abstract

The present study explored the attitudes toward individuals with bionic eyes and limbs, cochlear implants, and people with disabilities that imply using a wheelchair. Our sample consisted of 474 Romanian adults aged 18–61 (*M *= 27.56, *SD *= 11.80). Participants were randomly divided into five groups. They all filled scales related to personality characteristics, i.e., agreeableness, neuroticism, openness to experience, comprehension/intellectual efficiency, and previous contact with disability. Then, each group was presented with a vignette describing a character (wheelchair/bionic eye/bionic leg/cochlear implant/control group). Finally, they answered questions about their emotions, cognitions, and behaviors related to that context. Overall, our results suggested that higher agreeability, extraversion, openness to experience, intellectual complexity, and lower neuroticism were generally associated with more positive attitudes toward disability. When examining the differences in participants' emotions, cognitions, and behaviors depending on the target's characteristics, our results generally suggested that the most negative reactions were toward the character with a bionic eye. We discuss these findings considering their importance for shaping positive attitudes related to disability, especially related to the future technological advances in bionic devices.

## Introduction

As a result of an increasing elderly population and shifts in the demographic makeup of various cultures (1), disability has evolved into an inevitable facet of the human experience in multiple parts of the world. According to the World Health Organization (2), the development of a disability, which can take on a wide variety of forms, is the result of the interaction between people with a health condition and the personal and environmental elements they are exposed to. More than one billion individuals, which accounts for fifteen percent of the world's population, are living with a variety of disabilities, which results in a significant economic, societal, and medical burden around the globe (3). As a result, it encourages the general public to contemplate effective solutions for incorporating and supporting those with disabilities. In recent years, several countries have begun to construct social and rehabilitative plans to alleviate the burden and improve the well-being of persons with mental and physical impairments who live in their communities (4).

Appraising a person's attitudes regarding their disability can be multifaceted—either positive or negative, or a mixture of the two (5). Several studies have investigated the effects of various attitudes. For instance, positive social attitudes could make it easier for people to be accepted by their families, friends, and employers (6). In contrast, negative attitudes could result in low expectations, discrimination, and marginalization (7). More specifically, research demonstrated that negative attitudes of healthcare providers had been indicated as a barrier to the involvement of individuals with disabilities in activities such as physical and educational contexts (8). In light of the current state of affairs and the significance of one's mentality, the general public needs to be encouraged to reevaluate and improve their perspectives on individuals with disabilities to create a more accepting society.

Individualistic societies, which emphasize personal identity and aspirations, have more favorable attitudes toward people with disabilities than collectivistic societies [e.g., (9)]. However, prostheses alter social lives, according to previous studies. Westbrook et al. (10), for example, studied six Australian communities (Chinese, Italians, Greeks, Germans, Arabic speaking, and Anglo-Australians) and found that Germans (individualists) were the most accepting of amputees, while Greeks and Arabs (collectivists) were the least, most likely due to the associated cultural features. At the same time, in the past years, globalization increased cultural uniformity, making cultures less collectivistic (11).

## Technological evolution and bionic prosthetics

In recent years, technology has rapidly advanced to assist people with physical limitations. Prostheses and bionic hands gained widespread media and public attention. However, in Romania, the public health system does not cover the full costs of such protheses, which can be challenging for prospective users (12). Nevertheless, new questions mark specific research directions as the world progresses and people become increasingly familiar with the expanding technology for assisting people with different disabilities. These questions include: how and to what extent does artificial intelligence (AI) in bionic devices impact persons with disabilities? Is it possible that it also brings drawbacks besides the great benefits of specific devices such as cochlear implants or bionic hands and limbs? Kaplan (13) researched the cultural differences in AI perception and found that eastern cultures, such as the Japanese, respond more positively to various forms of AI than Western cultures. On the other hand, according to Hirai and Dautenhahn (14), people who live in western cultures tend to view AI as a competitor, as the function of technology is still regarded with suspicion and fear. Similarly, the Frankenstein Syndrome refers to these concerns triggered by the use of AI, including bionic prosthetics (15).

Jack E. Steele, the creator of the American television show “The Six Million Dollar Man and Bionic Woman,” is credited with being the first person to use the term *bionics*. The characters of the show were given superpowers using electromechanical implants. After that, this term became popular in various contexts, including television and literature (16). However, in the context of the terminology currently in use, it primarily refers to medical equipment that directly connects with the disabled individuals' remaining neurological or muscle systems (17). In other words, a bionic prosthetic includes a mechanical or electric part that directly stimulates a nerve or muscle, obtaining the same results as a functional anatomic organ or limb, in most cases replacing it.

Only a few studies explore the attitudes toward people with bionic prosthetics, especially within the Romanian culture. For example, Maftei and Oancea-Matei (18) investigated whether a bionic target would generate different reactions in sacrificial dilemmas compared to other targets. Their results suggested that children were more willing to sacrifice the bionic targets compared to non-disabled and or targets with a disability. Meyer and Asbrock (19) also explored how stereotypical attitudes toward people with disabilities are affected by bionic prostheses. According to their results, using technology can alter the negative stereotypes associated with persons with disabilities (e.g., people with disability are warm but incompetent). As Meyer and Asbrock (19) suggested, people might see individuals using these bionic devices (i.e., bionic arm and leg prostheses, exoskeletons, or retina implants) as more competent than those with physical disabilities in general. Additionally, those who use bionic prostheses are thought to have a warmer disposition than able-bodied people. However, when the term “cyborg” was used instead of “bionic prostheses,” people had the impression that cyborgs were intelligent but unfeeling, which made them appear menacing (19).

## Target characteristics, contact, personality, and the attitudes toward disability

Examining people's views toward disability is crucial for addressing issues of equity, acceptance, and inclusion of people with disabilities. Various scholars explored this subject, and their conclusions are quite similar. For example, in the comprehensive review of Freer (20), the results suggested that, generally, females hold more favorable attitudes than males, and knowing a friend or a family member with a disability increases the chances of having more positive attitudes, as well [a result also confirmed within the Romanian population by Maftei and Gherguț (21)]. Also, inclusive education seems to be associated with students' more positive attitudes toward disabilities [e.g., (22)].

When it comes to age, however, the findings are mixed. Some studies suggested that younger individuals have more positive attitudes toward disability [e.g., (23, 24)], while others suggested the opposite (25). Also, other scholars found no significant differences in this regard [e.g., (26)].

The finding that concerns the association between the previous contact with disability and attitudes is the one that has been spoken about the most frequently among all of the components that have been discussed. The length of time spent in contact with disabled persons, the regularity and quality of that contact, and having disabled friends, family members, or coworkers are all examples of the manifestations of contact. In the vast majority of studies [e.g., (27, 28)], researchers concluded that interacting with persons who have disabilities could result in more favorable attitudes about those individuals. This finding may be because more contact could assist in reducing fear and anxiety and produce a more balanced and realistic perspective of the functional capacity and ability of persons with disabilities (27).

Evidence also suggests that persons who interact with disabled people will view themselves as more valued in social life and therefore be less likely to approach disabled people in a hostile manner (29). On the other hand, it is important to point out that a negative attitude toward disabled individuals would prevail in the absence of any controls over the demographic characteristics and the level of contact. For example, when the contact quality is not particularly examined, increased exposure may suddenly lead to uncomfortable or unpleasant sentiments, and people may associate these negative experiences with the disabled persons themselves (30). Therefore, it is also essential to consider the contact's number and quality rather than focusing solely on the frequency of the interactions. Even if some scholars [e.g., (31)] have found no substantial difference between attitude and contact, the causes that lead to those findings are still explicable (i.e., the lack of planned relationships between students and disabled people or the low frequency of such relationships). As a result, prior knowledge of the disabled individual's condition may be required under specific contact situations to foster a more positive attitude toward impairment. The decreasing tension between groups and the establishment of an atmosphere that could not only diminish prejudice but also encourage constructive relationships in a more pleasurable manner hints at the existence of a virtuous cycle of beneficial outcomes (32).

In addition to the elements mentioned up until this point, it has been suggested that public opinion also relies on individual aspects of the disabled person, such as the type of disability (33). For example, people tend to have less of a positive attitude toward individuals with a more evident physical disability (34). Also, people tend to have more positive attitudes toward people who are blind and deaf compared to those who are paralyzed and intellectually disabled (35). In addition, people with facial anomalies are likely to be perceived less favorably than people with other types of physical disabilities (32). Finally, a series of psychological factors have also been examined by previous studies regarding attitudes toward disability. For example, Hellmich and Loeper (36) suggested a positive correlation between self-efficacy and disability views. Armstrong et al. (23) suggested that reduced contact anxiety and high empathy scores were associated with positive disability attitudes. Also, self-esteem predicts more positive attitudes toward sensory impairments but not physical limitations, according to de Laat et al. (35).

However, regarding personality characteristics and variables such as agreeability, extraversion, neuroticism, openness to experience (i.e., some of the big Five dimensions), and intellectual efficiency, the findings are scarce when exploring the attitudes toward disability. For example, Ekehammar et al. (37) suggested that generalized prejudice (racism, sexism, prejudice toward homosexuals, and intellectual disability) was indirectly affected by extraversion, openness to experience, and conscientiousness through right-wing authoritarianism, and by agreeableness through social dominance orientation, whereas neuroticism had no significant effect. Page et al. (38) suggested that higher levels of openness and agreeableness were significantly associated with positive attitudes toward intellectual disability. Similar findings were reported by more recent research, e.g., Himmelberger et al. (39), generally highlighting that high levels of openness, agreeableness, and extraversion, and low levels of neuroticism usually predict more positive attitudes toward disability.

Finally, the research regarding the link between comprehension/intellectual efficiency and attitudes toward disability is scarce. Some previous studies explored the link between emotional intelligence and prejudice (not specifically toward disability) and suggested a negative link between them [e.g., (40)]. Other studies suggested that (cognitive) intelligence test scores seem to be negatively related to racial prejudice, while self-perceived intelligence is positively related, highlighting the need to explore various mediating mechanisms (41). The meta-analysis conducted by Onraet et al. (42) also concluded that higher scores on intelligence tests predict lower levels of prejudice. One possible explanation in this regard is that individuals with lower intelligence are more inclined to adopt essentialist thinking (41), i.e., “the belief that members of a particular social category share a fixed underlying nature, or essence” (43).

## The present study

In the present study, we aimed to explore the attitudes toward individuals with bionic eyes and limbs, cochlear implants, and people with disabilities that imply using a wheelchair. Though previous scholars researched similar topics, the novelty of our approach is that (1) we used an experimental approach, addressing the related limitation mentioned by previous work, (2) we used vignettes depicting various impairments, including some that implied the use of bionic devices, and (3) we explored the role played by personality traits since the evidence in the area is scarce.

Based on the previous literature regarding the attitudes toward disability, bionic prosthethes, and the underlying psychological mechanisms, we assumed the following:

H1. The *bionic* targets would generate more negative attitudes compared to the non-bionic targets [e.g., (18, 19)].
H2. Higher agreeability, extraversion, openness to experience, comprehension/intellectual efficiency, and lower neuroticism would be significantly linked to more positive attitudes toward disability [e.g., (39)].
H3. More frequent previous contact with a disability would be significantly linked to more positive attitudes toward disability [e.g., (27, 28, 30)].

## Method

### Participants and procedure

Our sample was formed of 474 participants aged 18–61 (*M *= 27.56, *SD *= 11.80). Most participants were females (94.9%) with a high-school diploma (48.3%). Table 1 details the sample's characteristics. The only inclusion criterion was related to age (i.e., all participants had to be over 18).

**Table 1:** Sample characteristics (*N* = 474).

	*N* (%)
Gender (self-reported)	
Male	24 (5.1)
Female	450 (94.9)
Age distribution (Mdn = 20)	
>20	239 (50.4)
<20	235 (49.6)
Education	
High-school	229 (48.3)
Bachelor's degree	152 (32.1)
Master's degree	89 (18.8)
PhD	4 (0.8)

Data were collected through an online questionnaire and distributed via social media platforms and communication groups (Facebook, Instagram, Messenger, and WhatsApp), using the snowball technique (44), at the beginning of 2022. Many of the participants who filled out the form were students from the faculty where the authors were affiliated, who received course credits for their participation. All participants voluntarily participated in this study, and they were informed that the information they provided would remain anonymous and confidential and that they could retire from this study at any time. The time needed to complete the questionnaire was around 20 min. The research was conducted following the Helsinki Declaration ethical criteria and the ethical research requirements approved by the institutional board of the authors' institution.

### Measures

#### Interaction

We asked participants to assess the frequency of interaction with individuals with physical or intellectual disabilities in the past 12 months. The exact question was, *How often did you interact, in the past 12 months, with someone with a physical/intellectual disability?*. We used a 5-point Likert scale ranging from 1 (not at all) to 5 (very often).

#### Attitude toward disability

We used the 34-item Multidimensional Attitudes Scale Toward Persons With Disabilities [MAS scale; (6)]. The instrument comprises three factors (emotions, cognitions, and behaviors). Participants were presented with a vignette describing the situation. They were randomly assigned to one of the five groups based on the character's description. In these vignettes, a woman called Maria (the character from the vignette) “went to a coffee shop for lunch with some friends. A woman in a wheelchair (**Group 1**)/with a bionic eye, i.e., an electronic visual prosthesis (**Group 2**)/with a prosthetic bionic leg (**Group 3**)/with a cochlear implant (**Group 4**)/with no specific characteristics (i.e., **Group 5**—‘a woman’—control group), with whom Maria is not acquainted, enters the coffee shop and joins the group. Maria is introduced to this person, and shortly thereafter, everyone else leaves, with only Maria and the woman in the wheelchair remaining alone together at the table. Maria has 15 min to wait for her ride. Try to imagine the situation.” For groups 2, 3, and 4 (bionic eye/bionic leg/cochlear implant), the participants read a short description of these devices to ensure that everybody knew exactly what they meant. Then, they read the 34 items describing the emotions (e.g., tension/stress/pity/disgust), thoughts (e.g., She seems to be an interesting guy/girl/She looks like an OK person), and behaviors (Move away/Find an excuse to leave) that Maria might have expressed. Each dimension had questions that participants answered on a scale ranging from 1 (not at all) to 5 (very much). Cronbach's alpha-s (regardless of the experimental group) was 0.90 for the Emotions dimension, 0.92 for the Cognition factor, and 0.82 for the Behavioral dimension. Higher scores suggested more negative attitudes toward disability.

#### Agreeability

We used the 10-item Agreeability scale developed by Goldberg et al. (45) to measure agreeability. Example items included “I am interested in people” and “I take time out for others.” Higher scores suggested higher agreeability. Cronbach's alpha was 0.80.

#### Extraversion

We used the 10-item Extraversion scale developed by Goldberg et al. (45). Example items included “I am the life of the party” and “I feel comfortable around people.” Higher scores suggested higher extraversion. Cronbach's alpha was 0.78.

#### Neuroticism

We used the 10-item Neuroticism scale developed by Goldberg et al. (45). Example items included “I get upset easily” and “I become overwhelmed by events.” Higher scores suggested higher neuroticism. Cronbach's alpha was 0.91.

#### Openness to experience

We used the 10-item Openness to experience scale developed by Goldberg et al. (45). Example items included “I carry the conversation to a higher level.” and “I enjoy hearing new ideas.” Higher scores suggested higher Openness to experience. Cronbach's alpha was 0.72.

#### Intellectual efficiency

Finally, we used the 10-item Comprehension/Intellectual Efficiency scale developed by Goldberg et al. (45). Example items included “I have a rich vocabulary” and “I know the answers to many questions.” Higher scores suggested higher intellectual efficiency. Cronbach's alpha was 0.85.

A demographic scale assessed participants' age, gender, and education level.

## Results

### Preliminary analyses

We used the SPSS 26.0 program to analyze our data. We first computed the Skewness and Kurtosis values for the main variables to assess the normality of the distributions (46), and we further used parametric tests (see Table 2 for the descriptive statistics of the variables). We also computed the means and standard deviations for the main variables.

**Table 2:** Descriptive statistics for the main variables (overall sample, *N* = 474).

Variable	*M*	SD	Min	Max	Skewness	Kurtosis
Agreeability	43.66	5.18	22	50	−1.03	.91
Extraversion	32.02	6.85	12	48	−.02	−.32
Neuroticism	52.63	14.35	19	86	−.01	−.55
Openness to experience	39.86	5.34	25	50	−.48	−.33
Intellectual efficiency	38.95	6.22	20	50	−.31	−.28
Interaction (physical disability)	3.02	1.50	1	5	.07	−1.42
Interaction (intellectual disability)	3.06	1.57	1	5	−.02	−1.55
Attitude (MAS scale)	79.18	18.82	34	125	.16	−.59

### Correlation analyses

Next, we examined the associations between the main variables, considering the experimental group participants were distributed in (see Table 3).

**Table 3:** Zero-order correlations between the main variables (depending on the experimental group).

Variable	1	2	3	4	5	6	7	8	9	10
A Group 1 (wheelchair, *N* = 84)										
1. Agreeability	-			.						
2. Extraversion	.10	-								
3. Neuroticism	−.18	−.16	-							
4. Openness to experience	.32*	.40**	−.22*	-						
5. Intellectual efficiency	35*	.43**	−.28*	.54**	-					
6. Interaction (physical disability)	.06	.20	.08	.26*	.21*	-				
7. Interaction (intellectual disability)	−.03	−.07	.05	.08	−.05	.47**	-			
8. MAS—emotions	−.15	−.07	.25*	−.06	−.09	−.04	.15	-		
9. MAS—cognitions	−.27*	−.31*	.01	−.19	−.24*	−.13	.10	.25*	-	
10. MAS—behavior	−.32*	−.33*	.25*	−.20	−.18	.18	.32*	.38**	.27*	-
11. MAS—overall	−.30*	−.26*	.25*	−.17	−.20	−.01	.24*	.86**	.60**	.67**
B Group 2 (bionic eye, *N* = 75)										
1. Agreeability	-			.						
2. Extraversion	.18	-								
3. Neuroticism	.05	−.21	-							
4. Openness to experience	.50**	.18	−.05	-						
5. Intellectual efficiency	.31*	.18	−.25*	.46**	-					
6. Interaction (physical disability)	.07	.19	−.03	.14	.07	-				
7. Interaction (intellectual disability)	.23*	.31*	−.16	.08	.21	.56**	-			
8. MAS—emotions	−.12	.06	.14	−.09	−.14	−.04	−.14	-		
9. MAS—cognitions	−.41**	−.01	.12	−.37**	−.36**	.01	−.06	.22	-	
10. MAS—behavior	−.10	.07	.13	.01	.02	.08	.14	.34*	.20	-
11. MAS—overall	−.27*	.06	.18	−.19	−.22	.00	−.06	.84**	.59**	.64**
C Group 3 (bionic leg, *N* = 78)										
1. Agreeability	-									
2. Extraversion	.48**	-								
3. Neuroticism	−.20	−.39**	-							
4. Openness to experience	.50**	.20	−.18	-						
5. Intellectual efficiency	.54**	.49**	−.49**	.32*	-					
6. Interaction (physical disability)	.03	.18	−.27*	.08	.24*	-				
7. Interaction (intellectual disability)	.08	.16	.06	.17	.11	.36**	-			
8. MAS—emotions	−.07	−.05	.10	−.10	.14	.05	.14	-		
9. MAS—cognitions	.35*	−.24*	.05	−.19	−.20	−.06	−.21	.17	-	
10. MAS—behavior	−.20	.09	.04	−.15	.05	.13	.05	.41**	.28*	-
11. MAS—overall	−.25*	−.09	.10	−.19	.02	.05	.02	.82**	.59**	.72**
D Group 4 (cochlear implant, *N* = 82)										
1. Agreeability	-									
2. Extraversion	.26*	-								
3. Neuroticism	−.20	−.18	-							
4. Openness to experience	.36**	.22*	−.42**	-						
5. Intellectual efficiency	.39**	.21*	−.45**	.43**	-					
6. Interaction (physical disability)	.14	.29*	−.07	.23*	.30*	-				
7. Interaction (intellectual disability)	−.04	.17	.12	.11	.04	.45**	-			
8. MAS—emotions	−.06	−.05	.18	−.08	.09	−.02	−.00	-		
9. MAS—cognitions	−.43**	−.07	−.02	−.33**	−.19	−.19	−.15	.11	-	
10. MAS—behavior	−.13	.12	−.05	−.00	.03	−.23*	−.06	.24*	.10	-
11. MAS—overall	−.27*	−.01	.09	−.19	−.00	−.18	−.09	.82**	.53**	.58**
E Group 5 (control group, *N* = 155)										
1. Agreeability	-									
2. Extraversion	.41**	-								
3. Neuroticism	−.32**	−.37**	-							
4. Openness to experience	.37**	.41**	−.46**	-						
5. Intellectual efficiency	.34**	.41**	−.52**	.58**	-					
6. Interaction (physical disability)	.05	.13	−.14	.09	.14	-				
7. Interaction (intellectual disability)	.02	.07	−.11	.03	.19*	.55**	-			
8. MAS—emotions	−.08	−.25**	.29**	−.30**	−.10	.04	.04	-		
9. MAS—cognitions	−.41**	−.26**	.32**	−.31**	−.30**	−.05	−.11	.09	-	
10. MAS—behavior	−.25*	−.20**	.25**	−.19	−.15	.00	.15	.44**	.21*	-
11. MAS—overall	−.29**	−.33**	.40**	−.38**	−.23**	.01	.03	.86**	.50**	.69**

* *p *< .05.

** *p *< .001.

a. **Group 1** (i.e., the character in a wheelchair)

In Group 1, where participants were presented with the scenario involving a person in a wheelchair, we found significant associations between the overall attitudes toward disability and agreeability (*r* = −.30, *p *= .005), extraversion (*r* = −.26, *p* = .01), neuroticism (*r* = .25, *p* = .01), and the interaction with people with intellectual disabilities (*r* = .24, *p* = .02). Since higher scores on the MAS scale (i.e., attitudes toward disability) suggested more negative attitudes toward disability, our results suggested that the higher the agreeability, extraversion, and the lower the neuroticism, the more positive overall attitudes toward disability.

b. **Group 2** (i.e., the character with a bionic eye)

In Group 2, where participants were presented with the scenario involving a person with a bionic eye, we found a significant association between the overall attitudes toward disability and agreeability (*r* = −.27, *p *= .01). The other associations between the overall attitudes toward disability and the primary variables were non-significant. However, results also suggested that the cognitions dimension of the attitude toward disability was significantly associated with agreeability (*r* = −.41, *p *< .001), openness to experiences (*r* = −.37, *p *= .001), and intellectual efficiency (*r* = −.36, *p *= .001). Thus, our results suggested that the higher the agreeability, the more positive overall attitudes. Also, the higher the agreeability, Openness to experiences, and intellectual efficiency, the more positive cognitions related to disability.

c. **Group 3** (i.e., the character with a bionic leg)

In Group 3, where participants were presented with the scenario involving a person with a bionic leg, we found a significant association between the overall attitudes toward disability and agreeability (*r* = −.25, *p *= .02). The other associations between the overall attitudes toward disability and the primary variables were non-significant. However, results also suggested that the cognitions dimension of the attitude toward disability was significantly associated with agreeability (*r* = −.35, *p *= .002) and extraversion (*r* = −.24, *p *= .03). Thus, the higher the agreeability and extraversion, the more positive cognitions related to disability.

d. **Group 4** (i.e., the character with a cochlear implant)

In Group 4, where participants were presented with the scenario involving a person with a cochlear implant, we found a significant association between the overall attitudes toward disability and agreeability (*r* = −.27, *p *= .01). The other associations between the overall attitudes toward disability and the primary variables were non-significant. However, results also suggested that the cognitions dimension of the attitude toward disability was significantly associated with agreeability (*r* = −.43, *p *< .001) and Openness to experience (*r* = −.33, *p *= .002). Thus, the higher the agreeability and Openness to experience, the more positive cognitions related to disability.

e. **Group 5** (i.e., control group)

In Group 5, where participants were presented with a scenario involving a person with no specific characteristics, we found a significant association between the overall attitudes toward that specific character/situation and agreeability (*r* = −.29, *p *< .001), extraversion (*r* = −.33, *p *< .001), neuroticism (*r* = −.40, *p *< .001), openness to experiences (*r* = −.38, *p *< .001), and intellectual efficiency (*r* = −.23, *p *= .004). Similar patterns were observed for all the three dimensions explored (i.e., emotions, cognitions, behaviors) (see Table 3).

### Differences depending on the experimental group

Next, we explored the potential differences related to participants' overall attitudes toward disability depending on the experimental groups. We performed Anova One Way tests, and we used Bonferroni correction to control for the probability of committing a type I error. The results suggested significant differences, *F* (4; 469) = 2.47, *p *= .04. However, the only significant difference was found between the second (i.e., bionic eye) and the last group (control group), *M* dif = 7.94, *p* = .02. More specifically, participants from the control group (*M* = 76.40) reported significantly more positive attitudes than those from the bionic eye group (*M* = 84.34). When examining these means, we also observed that these were the groups with the most positive (Group 5) and most negative (Group 2) attitudes.

For a more comprehensive view of the emotions, cognitions, and behaviors related to disability, we repeated the Anova One Ways analyses for each of these three dimensions.

#### Emotions

Anova One Way test results suggested significant differences, *F* (4; 469) = 7.60, *p *< .001. We found a significant difference between the first (i.e., wheelchair) and the last group (control group), *M* dif = 6.03, *p* = .004. More specifically, participants from the control group (*M* = 35.60) reported significantly more positive emotions than those from the first group (*M* = 41.64). Also, we found significant differences between the bionic eye group (Group 2, *M* = 43.40) and the control group, *M* dif = 7.79, *p* < .001. More specifically, participants from the control group reported significantly more positive emotions than those from the bionic eye group. Similar patterns were observed between the bionic leg group (Group 3, *M *= 42.65) and the control group, *M* dif = 7.04, *p* = .001), and the participants from the fourth group (cochlear implant, *M* = 40.89) and the control group. In all cases, the more positive emotions were reported in the case of participants from the control group. Finally, when examining these means, we observed that the bionic eye group, Group 2, reported the most negative emotions (*M *= 43.40), and the control group was the most positive.

#### Cognitions

Anova One Way test results suggested no significant differences between the groups, *F* (4; 469) = 2.26, *p *= .06. However, when examining the means of the groups, we observed the group with the most positive cognitions was the cochlear implant group (*M *= 18.15), and the most negative was the control group (*M *= 20.80).

#### Behaviors

Anova One Way test results suggested significant differences, *F* (4; 469) = 3.96, *p *= .004. We found a significant difference between the first (i.e., wheelchair) and second groups (i.e., bionic eye group), *M* dif = −3.85, *p* = .001. More specifically, participants from the control group (*M* = 20.00) reported significantly more positive behaviors than those from the first group (*M* = 21.85). When examining the means of each group, we observed that the bionic eye group reported the most negative behaviors and the first group (i.e., wheelchair) the most positive behaviors (*M* = 18.00).

## Discussion

Our study investigated the attitudes toward people with bionic eyes and limbs, cochlear implants, and people with disabilities that imply using a wheelchair. Overall, our results suggested that higher agreeability, extraversion, openness to experience, intellectual complexity, and lower neuroticism were generally associated with more positive attitudes toward disability. However, when examining the differences in participants' emotions, cognitions, and behaviors depending on the target's characteristics, our results generally suggested that the most negative reactions were toward the character with a bionic eye. Thus, our primary assumptions were confirmed, highlighting the critical role of personality traits when discussing attitudes toward disability.

One of the interesting results of our study is related to the fact that intellectual complexity was associated with more positive attitudes toward disability. Previous studies also suggested that high intelligence test scores predicted lower prejudicial attitudes, though these studies did not specifically refer to disability [e.g., (40, 42)]. Thus, our study adds to the current literature exploring the link between intelligence and people's attitudes toward disability. Also, other scholars suggested that self-perceived intelligence was positively related to prejudice (41). In the present study, we did not measure participants' intelligence, as (40), but we asked participants to self-report their comprehension/intellectual complexity level. The different results that we obtained compared to those of De Keersmaecker et al. (41) might be related to the various psychological mechanisms that might also determine the attitudes toward disability, including cultural influences (47), peer norms (48), parental and social influences (49), moral values (50), or other related variables. Also, our measurements were different; we did not use the same scales, though the concepts share many similarities (51). Nevertheless, these findings highlight the need for further research in this area.

Another important finding in our study relates to the significant differences in participants' attitudes depending on the target's characteristics. We assumed that the *bionic* targets would be the ones toward which our participants would have the least positive attitudes, and our assumption was confirmed. This specific result aligns with previous findings in the area [e.g., (18)], highlighting the need for further programs to inform and explain bionic prosthethis and how they work, given that information is essential for shaping positive attitudes toward various disability and, implicitly, toward bionic devices and assistive technology, in general (52).

It is also important to acknowledge that, among the five experimental conditions, i.e., wheelchair/bionic eye/bionic leg/cochlear implant/control groups, the most negative attitudes were expressed toward the bionic eye character. That also raises the need for further, more complex, and in-depth research concerning the different perceptions and attitudes toward people with bionic prosthetics, depending on the type of bionic device used. Our results suggested that bionic eyes seem to be perceived more negatively than bionic legs, and this might be explained by the importance of perceived eye contact and facial expression (53). Nevertheless, future studies are needed to clarify the underlying psychological mechanisms better. Finally, though personality traits are rather unchangeable constructs, factual knowledge might be prone to intervention. We already know from previous studies the benefits of positive contact and information regarding disability (54), and future interventions might build on the current findings to build effective awareness and intervention strategies aimed to foster more positive attitudes.

A series of limitations need to be accounted for when interpreting our results. First, though we used an experimental approach, which adds value to our study, we used a relatively small sample of participants in each group. Future studies might benefit from using more extensive samples, and more heterogeneous in terms of age, education, and even disability status: it would be interesting to compare and assess the possible differences between these experimental groups by also diving them depending on disability status (i.e., with or without disabilities). Also, future studies might benefit from extending the target group to a larger number of people from different fields of activity. Next, though we provided short descriptions for each condition (e.g., for the bionic eye/bionic leg/cochlear implant), future studies using images instead of text might come to different conclusions.

Also another limitation is related to the self-reported measures of personality traits and intellectual complexity, which might have increased the desirability of the answers that participants gave. Also, we only used vignettes describing a female agent, and this type of vignette was used regardless of participants' gender. Though our sample was formed by 94.9% female participants, we must acknowledge this limitation, as well. Future studies might benefit from using gender-similar characters, to avoid any gender-based bias. Finally, future studies might benefit from exploring other variables that might account for significant variability when discussing the attitudes toward disability, and related bionic devices, such as cultural representations (55), media exposure and representations (56), social cognition (57), as well as specific knowledge of biotechnology, beliefs and support for more traditional values, and right-Wing authoritarianism (58).

The theoretical implications relate to the study of personality traits and self-reported intellectual complexity, the use of a multifaced scale for measuring the attitudes toward disability, and the experimental approach that allowed us to compare different targeted groups, including characters using assistive technology devices, i.e., bionic prosthetics. The practical insight brought by the present study is mostly related to the disability awareness programs that might be shaped to promote inclusive attitudes and positive views upon assistive technology, accounting for personality traits—in addition to other variables, as previous research suggested [e.g., (59)]. To conclude, we believe that, despite their limitations, the findings of our study are important considering their value for shaping positive attitudes related to disability, especially related to the future technological advances in bionic devices.

## Data Availability

The raw data supporting the conclusions of this article will be made available by the authors, without undue reservation.
